# Functional interactions between coat structure and colour in the determination of solar heat load on arid living kangaroos in summer: balancing crypsis and thermoregulation

**DOI:** 10.1007/s00360-024-01534-8

**Published:** 2024-02-09

**Authors:** Terence J. Dawson, Shane K. Maloney

**Affiliations:** 1 School of Biological, Earth and Environmental Sciences, University of New South Wales, Sydney, NSW 2052 Australia; 2 School of Human Sciences, University of Western Australia, Crawley, WA 6009 Australia

**Keywords:** Kangaroo fur, Solar radiation, Penetrance, Reflectance, Insulation, Crypsis

## Abstract

Interactions of solar radiation with mammal fur are complex. Reflection of radiation in the visible spectrum provides colour that has various roles, including sexual display and crypsis, i.e., camouflage. Radiation that is absorbed by a fur coat is converted to heat, a proportion of which impacts on the skin. Not all absorption occurs at the coat surface, and some radiation penetrates the coat before being absorbed, particularly in lighter coats. In studies on this phenomenon in kangaroos, we found that two arid zone species with the thinnest coats had similar effective heat load, despite markedly different solar reflectances. These kangaroos were Red Kangaroos (*Osphranter rufus*) and Western Grey Kangaroos (*Macropus fuliginosus*).

Here we examine the connections between heat flow patterns associated with solar radiation, and the physical structure of these coats. Also noted are the impacts of changing wind speed. The modulation of solar radiation and resultant heat flows in these coats were measured at wind speeds from 1 to 10 m s^−1^ by mounting them on a heat flux transducer/temperature-controlled plate apparatus in a wind tunnel. A lamp with a spectrum like solar radiation was used as a proxy for the sun. The integrated reflectance across the solar spectrum was higher in the red kangaroos (40 ± 2%) than in the grey kangaroos (28 ± 1%). Fur depth and insulation were not different between the two species, but differences occurred in fibre structure, notably in fibre length, fibre density and fibre shape. Patterns of heat flux within the species’ coats occurred despite no overall difference in effective solar heat load. We consider that an overarching need for crypsis, particularly for the more open desert-adapted red kangaroo, has led to the complex adaptations that retard the penetrance of solar radiation into its more reflective fur.

## Introduction

The fibres that cover the integument of mammals play many roles in the biology of those animals, from sensory input to camouflage and thermoregulation (see Dawson et al. 2014 for references). Insulation, which is fundamental to mammalian thermoregulation, is provided primarily by a relatively static air layer that is held in place by the fibres within the fur. This insulation restricts the flow of metabolic heat outwards in cold conditions (Scholander et al. 1950a, b, c; Hammel 1955) and the inward flow of environmental heat, especially when an animal is exposed to direct solar radiation (Schmidt-Nielsen et al. 1965; Dawson and Schmidt-Nielsen 1966). While the absorbance of solar radiation may benefit an animal when it is exposed to low environmental temperatures (Cooper et al. 2003; Wacker et al. 2016), for a diurnally active mammal that is exposed to high environmental temperatures, exposure to solar radiation can present a major thermal challenge. In some environments, radiation influx from the full solar spectrum (350–2100 nm) can exceed 1000 W m^−2^, with about half of that energy in the visible spectrum. Early researchers concluded that darker fur coats, with high absorbance at visible wavelengths, led to higher heat loads for animals than did lighter, more reflective, coats. That notion has changed considerably since the equations of Kovarik (1964) revealed that the interactions of solar radiation with animal coats could be more complex than black and white. Demonstration of the significance of such phenomena was made by Hutchinson and Brown (1969) for mammal fur and Walsberg et al. (1978) and Maloney and Dawson (1995) in avian feathers.

Only a proportion of the solar radiation that is incident on an animal’s coat is absorbed and converted into thermal energy at the coat surface. Of the radiation that is not absorbed at the coat surface, some is reflected to the environment, while a variable portion is reflected into the coat and absorbed deeper in the coat (Kovarik 1964; Hutchinson and Brown 1969). Radiation that is absorbed deeper in the coat thus produces a thermal load below the coat surface, closer to the skin. The resultant heat flows to either the skin or back to the environment in inverse proportion to the insulation in each direction. Studies of the coats of mammals (and a large bird) that would be impacted by this phenomenon show that the consequent solar heat load on the skin, the heat load from solar radiation (HL-SR), depends on the interactions between coat reflectance, insulation, and diverse coat structural characteristics such as fibre density, together with environmental features such as air movement (for references see Dawson and Maloney 2017). As our understanding of the involvement of colour and reflectivity in the heat load associated with solar radiation has continued to develop, it has emerged that when coat insulation exceeds some level (not yet defined) the HL-SR decreases and becomes progressively independent of coat colour. In the thick coats of the polar bear (*Ursus maritimus*) and the marsupial koala (*Phascolarctus cinereus*), which are white and dark grey, respectively, the proportion of the incident solar heat load that reached the skin surface (HL-SR) was similar and low in both species. The decrease in heat load with wind speed was also equivalent. The coat colour then seems to have little to do with thermoregulation but emerges as being primarily associated with crypsis, i.e., camouflage, with their reflectance to solar radiation closely matching that of their habitat (Dawson et al. 2014).

When insulation is low an alternate situation hypothetically arises. At an ideal surface without insulation, the HL-SR will be determined solely by the absorbance of the incident radiation. The thin, sparse coat of the numbat approaches this extreme (Cooper et al. 2003). Thus, the interaction between insulation and coat spectral characteristics in the determination of HL-SR will change as coat insulation changes. Additionally, as the insulating layer decreases, the penetrance of radiation will bring the radiation that is absorbed nearer to the skin, and “protected” from wind at the coat surface (Hutchinson and Brown 1969; Dawson et al. 2014). An unanswered question is, what is the lower level of insulation at which coat spectral characteristics begin to play a major role in the determination of effective HL-SR on mammals?

Dawson and Maloney (2017) examined the fur from the mid back and the hip from four species of kangaroo, including also the two colour morphs of the red kangaroo. Across these coats, there were notable variations in colour, reflectance, fur depth, and details of fur and fibre structure. The species were from a range of climatic zones, though at times, all face marked solar heat loads (Dawson 2012). As anticipated, coat depth (which was directly related to the level of insulation) had the strongest overall effect on HL-SR. However, there was complexity in the impact of coat reflectance. The thinnest coats had notably different reflectance, yet similar levels of HL-SR.

Dawson and Maloney (2017) considered that, in these species, fibre structural features impacted the penetration of solar radiation into the coat, and thus influenced the HL-SR. Such a prospect was especially curious in the case of two species, *Osphranter rufus*, the red kangaroo (red morph) and *Macropus fuliginosus* a grey kangaroo (western species). We have investigated further the heat flow patterns associated with solar radiation in the summer, back coats, of these kangaroos, focusing further on the impact of wind speed. We also look to the possibility that an overarching need for crypsis has led to the complex variability in our findings. The species appear to utilise differing behavioural strategies in their arid environment although various observations indicate some partitioning of their habitat (McCarron 1990; McCullough and McCullough 2000; Dawson 2012).

## Material and methods

Pelts were obtained from five male red kangaroos (*Osphranter rufus*) and five western grey kangaroos (*Macropus fuliginosus*) from the population overlap zone in north-western New South Wales (UNSW Arid Zone Research Station, Fowlers Gap). Note, the red kangaroo shows variation in coat colour, from ‘red’ (rusty brown) to ‘blue’ (light grey) and intermediate shades can arise. Males are mostly ‘red’ with the ‘blue’ morph only having a significant presence among females in the saltbush/bluebush shrubland in the semi-arid south of Australia (Dawson 2012). All the reds were of the “red” morph. The pelts were collected in the Austral summer (January and February). The pelts were salted and frozen in the field and then later thawed and stripped of subcutaneous fat and tanned in a salt/alum solution (Dimpel 1971) with pH adjusted to 4 during soaking and returned to 7 prior to removal, as outlined in Maloney and Dawson (1995). During drying each pelt was pegged so that its dimensions were the same before and after tanning. After processing, a region (about 260 mm diameter) was cut from the pelt in the central region of the back for measurement in the wind tunnel (see below) and small 20 mm diameter samples were taken immediately adjacent to that back region for the measurement of fur characteristics (see below).

Several of the smaller samples from each animal were used to measure a range of structural characteristics of the fur. Fur depth was determined at the natural lie of the pelt sample during measurements of thermal conductance in the wind tunnel (see below; Dawson and Brown 1970), as the average of four readings with a calibrated probe. The length of the fibres was measured as staple length by cutting a small batch of fibres from the sample at skin level. The fur fibres were orientated along a measuring scale and brushed lightly to achieve a lie parallel to the scale. Length was measured from the base to the tips of the guard hairs and separately as the length of the under-fur layer. Fibre diameter was measured on a microscope using a calibrated graticule. For each pelt, about 50 fibres were fixed on a microscope slide and measured. Fibre density was measured by punching (using a standardized punch) two samples of known surface area from the smaller samples. The punched samples were glued onto cards and the fibres on each of the punched sections were counted by removal with a fine scalpel under a dissecting microscope. Fur mass, mg cm^−2^ of skin, was determined by weighing the fur removed from a pelt sample of known area, approximately 30 × 10 mm. Fur bulk density, mg cm^−3^, was calculated by dividing fur mass by fur depth at the natural lie of the fur.

The spectral reflectance of three of the smaller samples from each animal was measured as described in Dawson and Maloney (2017). The spectral transmission of the glass used to make the wind tunnel was measured by the spectrophotometric facilities at the School of Optometry, University of New South Wales. These spectra were used to calculate the relative spectral distribution of light impinging on the samples in the wind tunnel, and thus the total reflectivity of the samples in the wind tunnel. Solar reflectance is reported, but the slightly different reflectance within the wind tunnel (adjusted for the power spectrum of the ARRI light and the glass of the tunnel) was used in the calculation of radiant heat load (HL-SR) and penetrance.

**Fig. 1:**
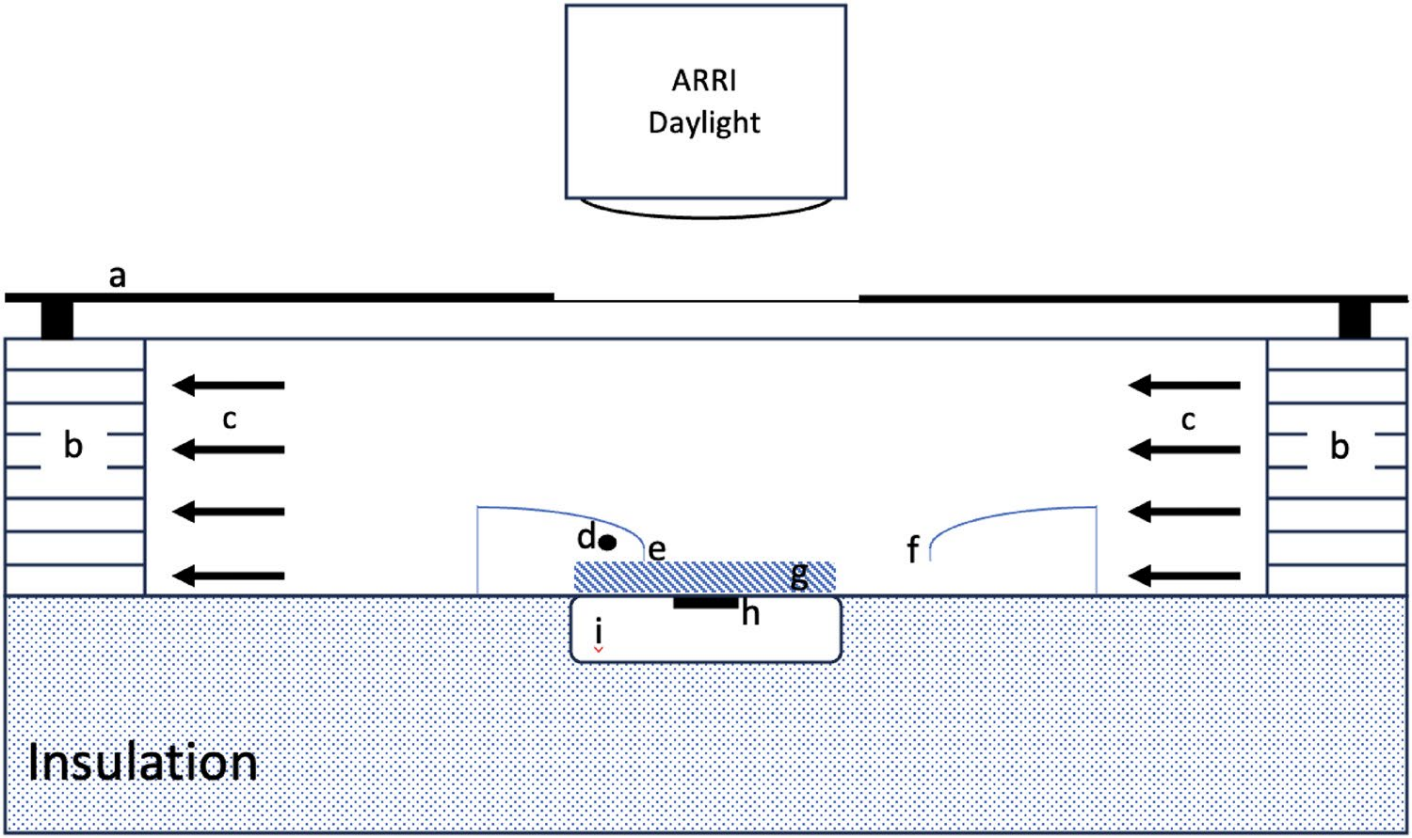
Diagram of the wind tunnel that was used in the experiments. **a** sheet of glass covered in aluminium foil except for a hole above the pelt sample, **b** flutes at inlet and outlet to encourage laminar flow through the wind tunnel, **c** wind direction driven by a rotary ventilator to the right of the flutes, **d** hot wire anemometer, **e** fine thermocouple measuring pelt surface temperature, **f** fine thermocouple measuring air temperature, **g** pelt sample, **h** heat flow transducers, **i** water filled hot plate connected to a circulating water bath. Other fine thermocouples, similar to e and **f**, were fed through a needle hole in the skin from below and bent level with the skin surface to measure skin temperature

The thermal properties of each pelt sample were measured in a laminar-flow wind tunnel utilising the techniques and equations of Hutchinson and Brown (1969), as modified by Maloney and Dawson (1995), and utilised also by Dawson and Maloney (2017). The thermal conductance (and, therefore, the insulation) was measured by mounting each sample on the upper surface of a temperature-controlled plate apparatus that was 260 mm in diameter with three 20 × 30 mm heat flux transducers embedded in its surface (Fig. 1). The samples were initially combed to reduce matting and then the samples were lightly smoothed in the direction of fibre lie to achieve an even surface. Each fur was orientated with fibre lying facing downwind. The water-filled plate was maintained at 38 ℃. Voltage output from the heat flux transducers (Thermonetics Corporation, USA), was logged via a DataTaker analog/digital (A/D) converter (Data Electronics Australia P/L, model 100F). Skin surface temperature (*T*_s_) was measured with two copper-constantan thermocouples (0.7 mm diameter) fed through oblique holes from beneath the skin. The temperature of the surface of the coat (*T*_e_) was measured with two similar thermocouples attached to flexible wires and bent into place for each pelt. Air temperature (*T*_a_) was measured with another two thermocouples placed in the air stream in front of the pelt sample, and plate temperature (*T*_p_) was measured with two thermocouples placed on the hot plate below the pelt sample. All thermocouples were referenced to a DataTaker isothermal block and logged on a personal computer via the A/D converter. The thermocouples were calibrated against a mercury-in-glass thermometer certified by the National Association of Testing Authorities, Australia, to an accuracy of ± 0.1 ℃. The heat flow transducers were calibrated using a certified thermal blanket of known thermal conductivity (National Institute of Standards and Technology, Gaithersburg, MD, USA). For each variable, the average of the two thermocouples, or three heat flux transducers, was used in the analysis.

The thermal conductance of each pelt was measured as a function of wind speed inside the wind tunnel. Wind speed was adjusted via a fan controlling airflow through the tunnel and was measured 20 mm above the sample with a Datametrics 810L thermoanemometer. Each sample was measured at wind speeds between 1 and 10 m s^−1^. The temperature of the air entering the tunnel (*T*_*a*_) was controlled at 20 ± 1 ℃ by placing the wind tunnel inside a temperature-controlled room. Thermal conductance (C) was calculated as C (W m^−2^ ℃^−1^) = *Q* / (*T*_*s*_ – *T*_*a*_), where *Q* is heat flow through the sample. Thermal insulation (m^2^ ℃ W^−1^) was calculated as the inverse of thermal conductance. The contribution of the air boundary layer to total insulation was obtained from calculations of the conductance of the fur itself, *C*_*P* =_ *Q* / (*T*_*s*_ – *T*_*e*_), and the conductance of the air boundary layer, *C*_a_ = *Q* / (*T*_*e*_ – *T*_*a*_).

The impact of solar radiation on heat exchange in the coat was examined by repeating the experiments with around (but known accurately) 600 W m^−2^ of short-wave radiation incident on the sample (the incident radiation intensity varied slightly with the depth of each coat because the surface of deeper coats was closer to the lamp). Radiation was supplied by an ARRI spotlight (ARRI Daylight 575 W). The relative spectral distribution of radiation from this lamp is similar in the visible and near-infrared part of the solar spectrum (Maloney and Dawson 1995). To minimise heating of the wind tunnel surrounding the sample, and transmission of infrared radiation to the sample from the hot lamp body, a piece of glass, covered in aluminium foil except for a hole allowing light to penetrate around the sample, was placed between the lamp and the wind tunnel. The lamp and second sheet of glass were cooled by forced convection. Incident radiation intensity was measured with a CSIRO SRI4 radiometer at a range of levels above the plate, appropriate for the range of depths of the pelts, generating a curve of intensity at the coat surface versus coat height. The radiation intensity at the level of the top of each sample was determined from that relationship. The proportion of the incident radiant heat load that acted as a heat load at the skin surface (PHLR) was calculated as:

$${\text{PHLR }} = \, \left[ {\left( {{\text{HF}}_{{\text{without radiation}}} } \right) \, - \, \left( {{\text{HF}}_{{\text{with radiation}}} } \right)} \right] \, /{\text{ incident radiation}},$$

where HF was heat flow through the sample.

Radiation that is incident on a coat surface can be either reflected or absorbed and converted to heat. The heat gain from solar radiation can be described using a model that was developed by Hutchinson and Brown (1969) from the equations of Kovarik (1964). Incident radiation that is not absorbed can be reflected either back to the environment (net reflection, the visible portion of which is perceived as animal colour) or deeper into the coat, where it is absorbed and converted into heat. This forward reflection manifests as the penetrance of radiation. In this model, it is assumed that there is an average depth to which solar radiation penetrates before it is absorbed, indicated as a single layer, *z*, such that the heat load resulting from this average penetration equals the heat load experienced with non-localised absorption (Hutchinson and Brown 1969). The heat resulting from the absorption of solar radiation at z flows either to the environment or to the skin, in inverse proportion to the insulation in each direction. Because the insulation of the air boundary layer, and at high wind speeds of the coat itself, decreases with an increase in wind speed, the fate of this absorbed heat thus also depends on the wind speed. The actual heat load from solar radiation at skin level (HL-SR), which impacts on the body, is then given by:

$$HL - SR \, = \, RA \, \left( {I_{z} + \, I_{e} } \right) \, / \, \left( {I_{c} + \, I_{e} } \right),$$

where *R* is the intensity of radiation incident on the surface of the coat, *A* the coat’s absorbance, *I*_*c*_ is the insulation of the coat, *I*_*z*_ is the insulation of the coat between the point of absorption and the coat’s surface, and *I*_*e*_ = the insulation of the air boundary layer. If HL-SR is expressed as a % of incident radiation, then:

$$\% {\text{HL}} - {\text{SR }} = \, \left( {{\text{HL}} - {\text{SR }} \times \, 100 \, / \, R} \right) \, = \, \left[ {A \, \left( {I_{z} + \, I_{e} } \right) \, / \, \left( {I_{c} + \, I_{e} } \right)} \right] \, \times 100$$

We measured all these components except *I*_*z*_, and so calculated *I*_*z*_, the average depth of penetration of incident solar radiation.

The characteristics of the coat were compared between the species using student’s *t* test. The effect of wind speed was compared between species using a general linear model (using the package lmer in R studio), after percentage and proportional variables had been arcsine transformed. Initially, a linear model was run using just species and wind speed (null) and then the same data were compared with individuals included as a random factor. The same analysis was then conducted using a second-order polynomial model (null and with the individual included as a random factor). For all but one of the variables that we tested, the polynomial model provided a significantly better fit to the data than did either the linear model or a null polynomial model, and so the polynomial model was used to analyse all of the data. For the insulation provided by the fur as a proportion of the total insulation, the polynomial model provided the same explanatory power as the linear model. To retain consistency in analysis, the polynomial model was used to analyse the insulation provided by the fur as a proportion of the total insulation. When differences between species with wind speed were indicated by the GLM, a post-hoc Tukey HSD test was used to compare the species at each wind speed.

## Results

While some of the morphological characteristics of the fur were similar between the red kangaroos and the grey kangaroos, several aspects differed markedly between the two species (Table 1). Coat depth did not differ significantly between the species (*t*_8_ = 1.10, *P* = 0.30), despite overall fur length (*t*_8_ = 3.33, *P* = 0.01), and under fur length (*t*_8_ = 6.68, *P* = 0.0002), being longer in the greys. The discrepancy can be explained because the angle that fibres lay relative to the skin was shallower in the greys (*t*_8_ = 2.81, *P* = 0.023). The fibre density was significantly higher in the red kangaroos (*t*_8_ = 6.60, *P* = 0.0002), although the fibre diameter was the same as in the greys (*t*_8_ = 1.56, *P* = 0.16). The fur mass (*t*_8_ = 0.16, *P* = 0.88) and fur bulk density (*t*_8_ = 1.01, *P* = 0.31) also do not differ between the species.

**Table 1:** Physical features of the back fur of two arid living kangaroos, the red kangaroo (*O. rufus*) and the western grey kangaroo (*M. fuliginosus*)

Parameter	Units	Red kangaroo	Grey kangaroo	Significance
Coat depth	mm	8.6 ± 1.3	10.0 ± 0.5	ns
Fibre density	fibres/mm^2^	44.4 ± 2.8	20.8 ± 2.2	***
Fibre diameter	μm	23.3 ± 1.0	25.2 ± 0.7	ns
Fur length	mm	18.1 ± 2.1	27.8 ± 2.0	*
Under fur length	mm	10.3 ± 0.9	19.0 ± 1.0	***
Fur mass	mg/cm^2^	19.8 ± 1.6	20.2 ± 2.4	ns
Fur bulk density	mg/cm^3^	24.8 ± 3.2	20.4 ± 2.4	ns
Fibre angle	degrees	29.3 ± 2.2	22.0 ± 1.4	*

Values are mean ± s.d. *N* = 5

Between species significant differences (*t* test)

^*^P < 0.05

^**^P < 0.01

^***^P < 0.001

**Fig. 2:**
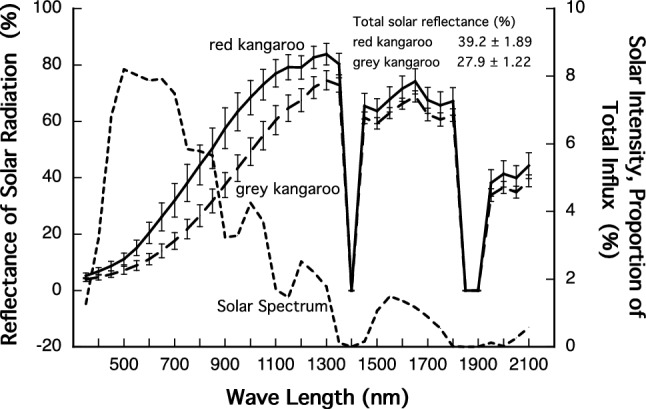
Reflection of radiation across the solar spectrum (350–2100 nm) from the back fur of two arid-zone living kangaroos, the red kangaroo (*O. rufus*), solid line, and the grey kangaroo (*M. fuliginosus*), long dashes. Values at measured wavebands are mean ± SD; *N* = 5. Also shown is the solar intensity across these wavelengths (short dashes). The spectrum of the ARRI lamp is similar to the solar spectrum up to 1100 nm, but above that, there is no power in the spectrum of the lamp (a comparison is given in Fig. 2 in Maloney and Dawson [1995])

Within the visible spectrum (380–700 nm) the back fur of the red kangaroos was significantly more reflective than was the back fur of grey kangaroos (Fig. 2). The integrated total reflectance across the *entire* solar spectrum was significantly higher in the red kangaroos than the grey kangaroos (*t*_8_ = 5.01, *P* = 0.001), being 39.2 ± 1.89% for the red kangaroo and 27.9 ± 1.22% for the grey kangaroo (Fig. 2). The integrated reflectance within the wind tunnel also differed between the species (*t*_8_ = 5.15, *P* = 0.0008).

**Fig. 3:**
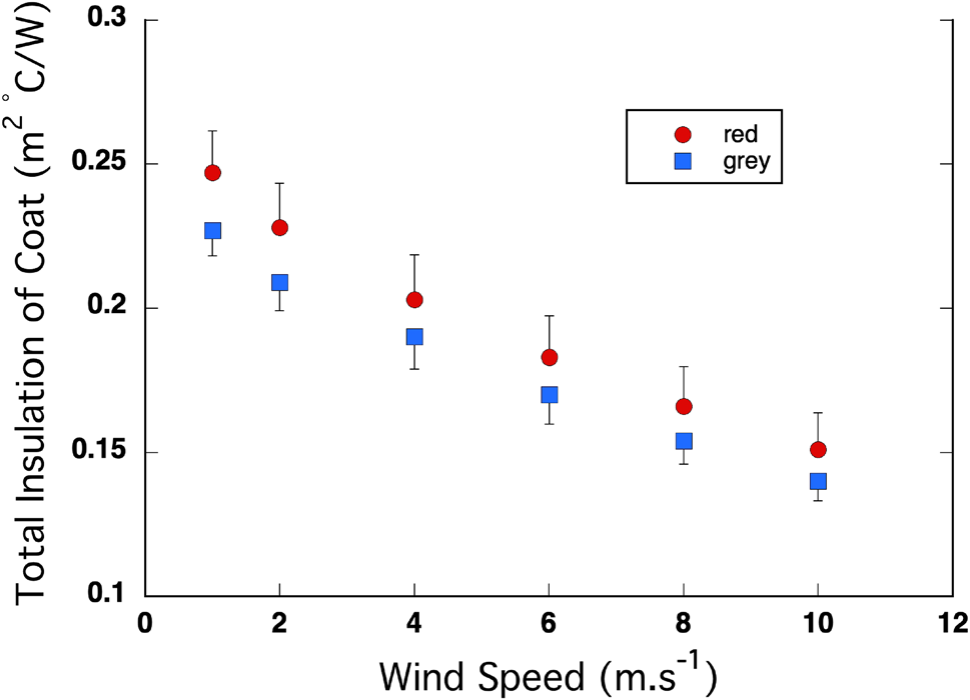
Total insulation (fur + air boundary layer) of the coats of the red kangaroo (*O. rufus*) and the grey kangaroo (*M. fuliginosus*) decreased significantly with increasing wind speed. However, there was no significant difference in the response between the two species. *N* = 5 for each species

The total insulation (fur + air boundary layer) decreased significantly with wind speed in both species (Fig. 3). The intercept was significantly positive (*P* = 4 × 10^–9^) but did not differ between the species (*P* = 0.23). The coefficient for *x*^2^ was significantly positive (*P* = 0.0002), indicating that the rate of decline in insulation decreased as wind speed increased, while the coefficient for *x* was significantly negative (*P* > 10^–14^) indicating an inverse relationship, but neither of the coefficients differed between the species (*P* = 0.24 and 0.46, respectively). As a result, there was no difference in total insulation between the species at any wind speed.

**Fig. 4:**
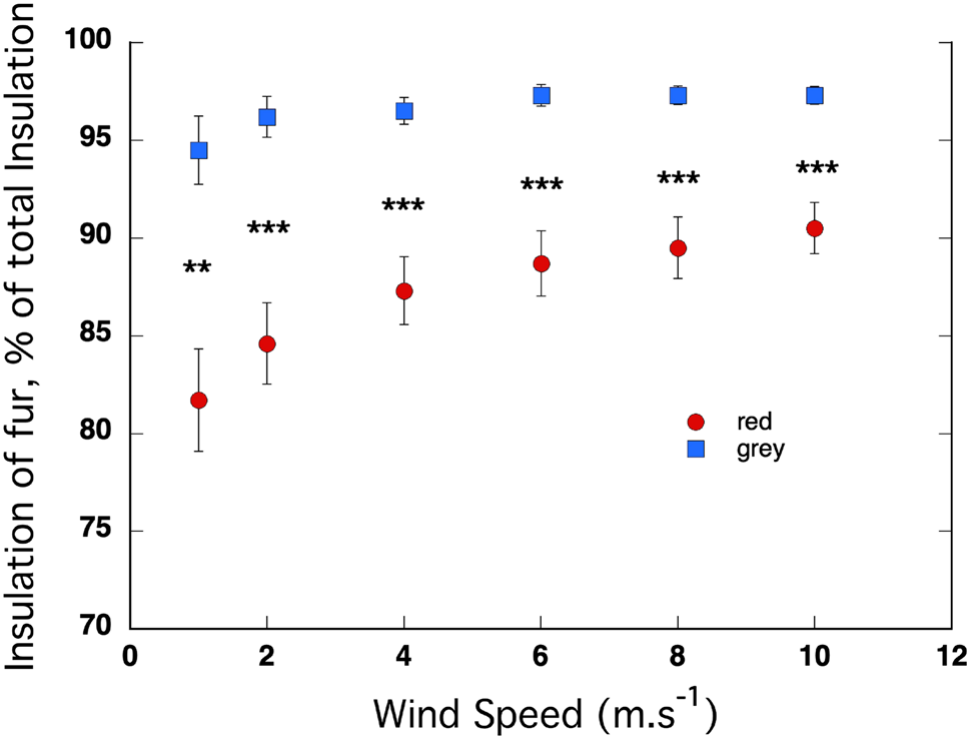
The percent of total insulation provided by the fur was influenced by wind speed in the coats of the red kangaroo (*O. rufus*) and the grey kangaroo (*M. fuliginosus*). Overall, the insulation provided by the coat of the grey kangaroo was a significantly higher proportion of the total insulation than it was in the red kangaroo. The pattern of change with wind speed also differed between the species. *N* = 5 for each species

The insulation provided by the fur as a proportion of the total insulation increased significantly with wind speed, beginning with a positive intercept (*P* < 10^–16^) that was significantly higher in the red kangaroos than the grey kangaroos (*P* = 2 × 10^–5^, Fig. 4). The coefficient for *x*^2^ was significantly negative (*P* = 0.002) and did not differ between the species (*P* = 0.22), indicating that the rate of increase decreased as wind speed increased. The coefficient for x was significantly positive (*P* = 10^–6^), indicating a positive relationship with wind speed, and the coefficient for the red kangaroos was significantly higher than the coefficient for the grey kangaroos (*P* = 0.02), indicating that the insulation provided by the fur changed more with wind speed in the red kangaroos than it did in the grey kangaroos. The insulation provided by the fur as a proportion of the total insulation was significantly higher in the grey kangaroos than in the red kangaroos at every wind speed (ranging from *P* = 0.004 at 1 m s^−1^ to *P* = 0.001 at 10 m s^−1^).

**Fig. 5:**
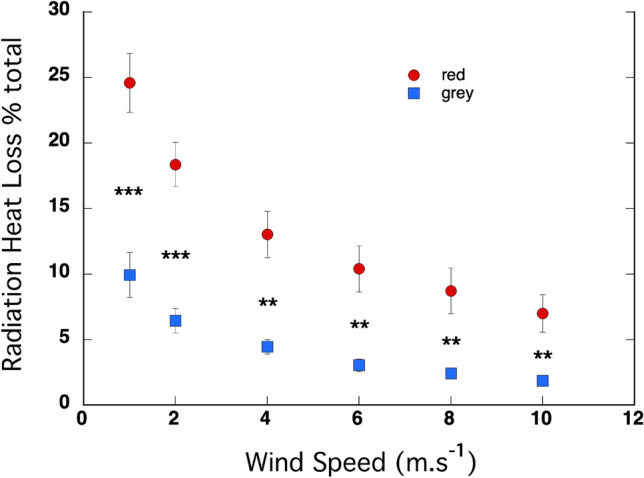
Radiant heat loss*,* as a % of total heat loss, varied with wind speed in the coats of the red kangaroo (*O. rufus*) and the grey kangaroo (*M. fuliginosus*). Overall, levels were higher in the red kangaroo. For both species, the rate of change in HLR% decreased with increasing wind speed with the radiant heat loss as a proportion of the total heat loss being significantly lower in the grey kangaroo than it was in the red kangaroo at every wind speed. *N* = 5 for each species

The proportion of the heat that was lost from the pelts of the two species by radiation decreased as the wind speed increased, indicating that more heat was lost by convection as the wind speed increased (Fig. 5). The intercept was significantly positive (*P* < 10^–11^) and significantly higher in the red kangaroos than the grey kangaroos (*P* = 10^–6^). The coefficient for *x*^2^ was significantly positive (*P* < 10^–7^) indicating that the rate of decline in heat loss by radiation decreased as wind speed increased, and the coefficient was higher in the red kangaroos than the grey kangaroos (*P* = 0.04) indicating that the rate of decline was higher in the red kangaroos than in the grey kangaroos. The coefficient for x was significantly negative (*P* > 10^–13^) and more negative in the red kangaroos than the grey kangaroos (*P* = 0.0004), again indicating a steeper decline in the red kangaroos. The radiant heat loss as a proportion of the total heat loss was significantly lower in the grey kangaroos than the red kangaroos at every wind speed, ranging from *P* = 0.0008 at 1 m s^−1^ to 0.008 at 10 m s^−1^.

**Fig. 6:**
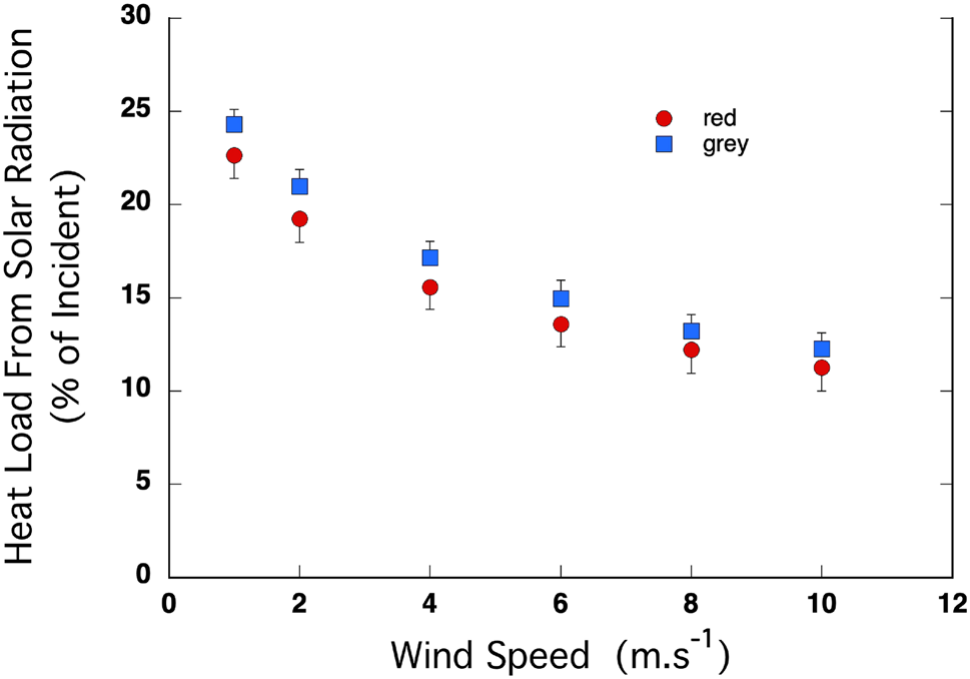
The proportion of the radiant heat load that impacted as a heat load on the skin decreased with wind speed in the coats of the red kangaroo (*O. rufus*) and the grey kangaroo (*M. fuliginosus*). There were no significant differences between species at any wind speed or the rate of change. *N* = 5 for each species

The percent of incident radiation that acted as a heat load at the skin level decreased significantly with wind speed in both species (Fig. 6). The intercept was significantly positive (*P* < 10^–9^) and did not differ between the species (*P* = 0.28). The coefficient for *x*^2^ was significantly positive (*P* < 10^–9^) indicating that the rate of decline in HL-SR decreased as wind speed increased, and that rate did not differ between the species (*P* = 0.91). The coefficient for *x* was significantly negative (*P* < 10^–15^) and did not differ between the species (*P* = 0.85). As a result, there was no difference in HL-SR between the species at any wind speed.

**Fig. 7:**
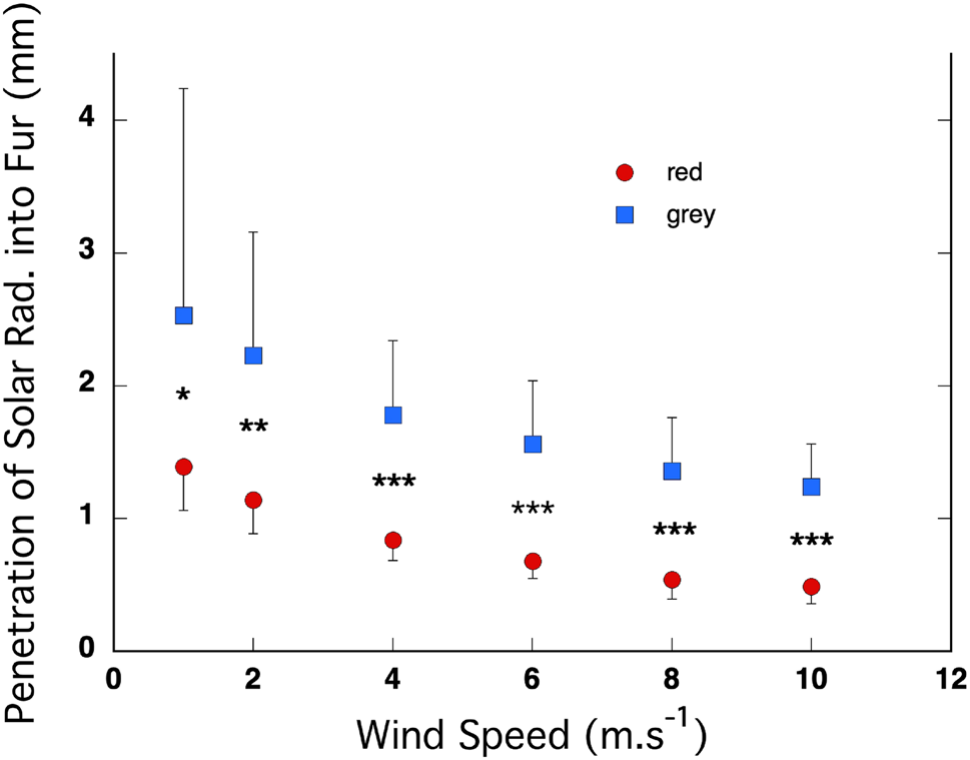
Solar radiation penetrated less into the furs of the red kangaroo (*O. rufus*) than it did into the fur of the grey kangaroo (*M. fuliginosus*). Overall, the penetration of radiation decreased with increasing wind speed in a similar manner in both species. There was a significant difference between the species at every wind speed. *N* = 5 for each species

The penetration (in mm) of solar radiation into the fur decreased significantly with wind speed in both species (Fig. 7). The intercept was significantly positive (*P* < 10^–8^) and significantly higher in the grey kangaroos than in the red kangaroos (*P* < 10^–4^). The coefficient for x^2^ was significantly positive (*P* = 0.03), indicating that the rate of decline in penetration decreased as wind speed increased, and did not differ between the species (*P* = 0.74). The coefficient for x was significantly negative (*P* = 0.0004) and did not differ between the species (*P* = 0.40). The penetrance (in mm) of solar radiation into the fur was significantly higher in the grey kangaroos than the red kangaroos at every wind speed, ranging from *P* = 0.016 at 1 m s^−1^ to 0.0006 at 10 m s^−1^.

**Fig. 8:**
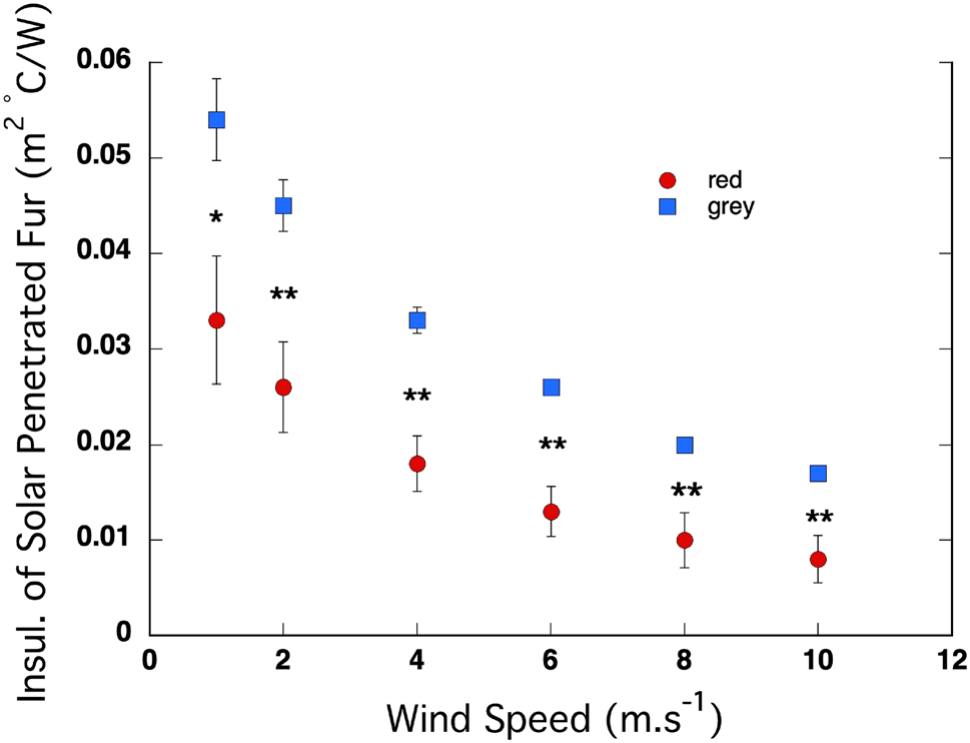
The penetrance (in terms of insulation) of solar radiation into the fur of the red kangaroo (*O. rufus*) and the grey kangaroo (*M. fuliginosus*) decreased with wind speed in a curvilinear manner, with the rate of change in penetration decreasing similarly with increasing wind speed in the two species. There was a significant difference in the level of penetration between the species at every wind speed. *N* = 5 for each species

When the penetration of radiation into the fur is expressed in terms of insulation value (that is, the insulation provided by the coat between the point of absorption, *z*, and the coat surface) that penetration decreased significantly with wind speed (Fig. 8). The intercept was significantly positive (*P* < 10^–10^) and was significantly higher in the grey kangaroos than the red kangaroos (*P* < 10^–4^). The coefficient for x^2^ was significantly positive (*P* = 0.005), indicating that the rate of decline in penetration decreased as wind speed increased, and did not differ between the species (*P* = 0.51). The coefficient for x was significantly negative (*P* < 10^–4^) and did not differ between the species (*P* = 0.15). The penetrance (in terms of insulation) of solar radiation into the fur was significantly higher in the grey kangaroos than the red kangaroos at every wind speed, ranging from *P* = 0.025 at 1 m s^−1^ to *P* = 0.009 at 10 m s^−1^.

## Discussion

Previously we examined the thermal characteristics of summer coats of species of kangaroo that inhabit diverse environments (Dawson and Maloney 2017). An area of focus was on mechanisms that could ameliorate the impact of the solar thermal loads that can exist in hot environments. Two species that may overlap in arid zones, the red kangaroo (*O. rufus*) and the western grey kangaroo (*M. fuliginous*) differed in their adaptive responses. The red kangaroos had a higher coat reflectance and, apparently, structural modifications of the fibres that helped to limit the heat load from solar radiation. On the other hand, the grey kangaroos had coats with similar insulation to the reds, but a relatively low reflectance and no evidence of modification to the fibres. But HL-SR was only slightly higher than it was through the coat of the red kangaroo when examined at low wind speeds. This finding was unexpected, given that grey kangaroo coats absorbed nearly 25% more radiation. We have used the coats of these two kangaroos to further explore the complexities of heat flow in mammalian fur, particularly where levels of insulation are relatively low. Our results relate to measurements made with and without a solar heat load and at a range of ecologically relevant wind speeds. The effects of wind speed differentially effect various coat characteristics and have provided insight into the processes of heat flow into animal coats.

Although the total insulation of the back coats of the red kangaroos and grey kangaroos decreased markedly with increasing wind speed, the initial similarity between the species at low wind speed was maintained across a tenfold increase in wind speed (Fig. 2). That is a curious result given their marked differences in fur structure (Table 1). The coats of the red kangaroo have a much higher fibre density, while the coats of grey kangaroos have longer fibres, both for the guard fibres and the under-fur fibres, which results in equivalence in bulk and depth and hence insulation at low air movement (Dawson and Maloney 2017). Given that the fibre diameter, which is known to influence insulation when wind speed increases, at least in alpacas (Moore et al. 2011), was the same in the two species of kangaroo, it is a logical prediction that a denser coat would maintain a more stable air layer when wind speed increases. But insulation in the red kangaroos with their denser coat, decreased the same with an increase in wind speed as it did in the grey kangaroos. Insight into this situation can be provided by the contribution of the coats alone to the total insulation over a range of windspeeds (i.e., absent the impact of wind on the air boundary layer) (Fig. 3). In red kangaroos, the coat alone contributed much less to the total insulation than it did in grey kangaroos, and that pattern was maintained across the range of wind speeds. The other component of insulation, the air boundary layer, was thicker in the red kangaroo than it was in the grey kangaroo, and even though the insulation provided by the air boundary layer decreased more in the reds than it did in the greys with an increase in wind speed, the layer was maintained better in the reds when the wind speed increased. This effect is likely related to the higher density of shorter fibres in the fur of the red kangaroos and additionally, that the fibres in red kangaroo coats are crimped or wavy (Dawson and Maloney 2017) both of which likely contributed to the stability of their air boundary layer when wind speed increased.

A pattern that emerged in the various routes of heat loss through the coats of red kangaroos and grey kangaroos further suggests that the structural differences in the coats of the two species contribute adaptively to their thermoregulation. In the coats of both species heat loss by radiation was quite a small component of the heat loss, compared to losses by convection and conduction. But heat loss by radiation was significantly higher from the coat of the red kangaroo than it was from the coat of the grey kangaroo. The difference was the largest at low wind speed, but the difference was maintained across wind speeds (Fig. 4), thus confirming that the structural integrity of the coat of red kangaroos contributed to a lower heat loss by convection than in the greys. With a thicker air boundary layer in the red kangaroos, the surface temperature of the coat was maintained closer to skin temperature than it was in the grey kangaroos, resulting in more heat loss by radiation from the coats of the red kangaroos.

The fibres that make up the coat of red kangaroos, while being shorter and denser, were more upright (the angle of lie was higher) than the fibres in the coats of grey kangaroos. In the coat of the grey kangaroo the longer, sparser, fibres overlay each other. The result was an equivalent fur depth and fur bulk density, and thus insulation. The overall insulation was the same despite differing patterns of heat flow in the furs of the two species. Whether, in the natural environment, such patterns of heat flow could have different functional effects, for example in facilitating or inhibiting surface evaporative heat loss, is conjectural.

In addition to playing a major role in insulation against heat loss, the coat of mammals has a specific role in the amelioration of heat loads associated with incident solar radiation, which can be extreme during summer days in the habitat of these kangaroos (Dawson 1972). On a bare surface, the heat load from solar radiation is a direct function of the colour of the surface. In that sense, the coat of the more desert-associated red kangaroo (light brown) was markedly more reflective (39.2 ± 1.9%) than was the dark grey coat of the grey kangaroo (27.9 ± 1.2%) (Table 1; Fig. 1; Dawson and Brown 1970; Dawson and Maloney 2017). However, we found no significant differences between these kangaroos in the proportion of the incident solar load that acted as a heat load at the skin (HL-SR) (Dawson and Maloney 2017; Fig. 5), even when HL-SR declined markedly with increasing wind speeds (Fig. 5). Why and how does that occur? Such results highlight the complexity of interactions between coat characteristics on the fate of incident radiation within the coat.

The influence of coat depth, fibre diameter, fibre density, fibre angle, and reflectance as predictors of HL-SR at low wind speed in the coats of four kangaroo species was examined by Dawson and Maloney (2017). Broadly, across those coats, reflectance had the strongest impact on HL-SR and the effect seemed intuitive, that is, as reflectance increased, HL-SR decreased. Similarly, as the depth of the coat increased, HL-SR decreased. In coats that are deeper than those of kangaroos, very little heat from solar radiation reaches the skin, irrespective of fur colour, as noted in polar bears (*Ursus maritimus*) and the marsupial koala (*Phascolarctus cinereus*) by Dawson et al. (2014). With a coat depth of 29.8 mm (polar bear) and 26.5 mm (koala), the coats of those animals were considerably deeper than the red (8.6 mm) and grey kangaroos (10.0 mm) in the present study, and so it was perhaps not surprising that HL-SR was still impacted by coat colour and reflectivity in the kangaroos. The hypothesised limit where HL-SR becomes independent of colour is apparently beyond 10 mm. In the study of Dawson and Maloney (2017) most other structural features of coats showed no overall effect on HL-SR; however, some did influence the penetration of solar radiation into the coat. As fibre density increased, solar penetration decreased, and as fibre angle increased, penetrance increased. Can these interactions help to explain the similarity in HL-SR in the back coats of the red kangaroo and the grey kangaroo, despite absorption levels being, respectively, 61 and 72%?

The way and the depth that radiation is absorbed in the fur determines the heat load that reaches the skin. Radiation incident on the coat surface that is not reflected back to the environment is either absorbed at the surface or reflected deeper into the coat. In turn that forward scattered radiation can be reflected back toward the coat surface, and perhaps ultimately to the environment, or it may be absorbed within the coat and converted into thermal energy. The fate of radiation that is incident on an animal coat can be described using a simple model that was developed by Hutchinson and Brown (1969) from the equations of Kovarik (1964). They described that penetration is known to be influenced by the reflectivity of individual coat elements and the density of those elements. The key to the model is that it assumes an average depth to which solar radiation penetrates before being absorbed (see Appendix Fig. 9). Radiation is likely absorbed in all levels within the coat, but the heat load on the skin that results from the average penetration is equal to the heat load that is experienced with non-localised absorption (Hutchinson and Brown 1969). That average level is designated as z, from where the heat that is produced from the absorption of solar radiation flows either to the environment or to the skin, in inverse proportion to the insulation in each direction. Thus, the heat load on the skin depends on z and also on factors that affect the insulation through the fur from z to the skin or to the environment. One of those factors is the air boundary layer, which contributes to the insulation from z to the environment. Forced convection due to wind markedly reduces the air boundary layer and hence the effective insulation from z to the environment.

When the penetrance model was developed it was thought that a darker coat, with a high absorbance of radiation, would be made from individual elements that also had a high absorbance of radiation. Following that logic, a darker coat should absorb radiation closer to the surface of the coat, and have a small z. In contrast, while coats with elements that reflect more radiation would absorb less radiation overall, but the radiation that was absorbed would be absorbed deeper in the coat because of forward scattering from the reflective elements. That is, lighter-coloured coats should have a deeper z. Some of the early studies that tested the predictions of the model found that notion to hold true (Hutchinson and Brown 1969; Walsberg et al. 1978). Given the higher reflectivity of the coat of the red kangaroo, it might be expected to have a deeper z than the coat of the grey kangaroo*.* But that is not what we found, and in fact the reverse was apparent. The z layer in the red kangaroo was closer to the surface of the fur than it was in grey kangaroos*,* and thus more of the heat from the absorbed solar radiation flowed outward to the environment in the red kangaroo. What appears to be the basis of this effect is the markedly higher fibre density, in combination with upright, crimped fibres, in the coat of the red kangaroo (Dawson and Maloney 2017). Crimping will increase the probability that an incident photon will strike a fibre within the coat, limiting the “free path” to deeper penetration. The same logic applies to fibre density, more fibres increase the probability that an incident photon will strike a fibre within the coat. The high fibre density that we measured in the red kangaroos is not seen in other species of kangaroo, even those that are also in the genus *Osphranter*, the wallaroos and the antilopine kangaroo, which have fur densities closer to those of the western grey kangaroo and its close relative the eastern grey kangaroo (*M. giganteus*), a mostly forest and woodland inhabitant (Dawson and Brown 1970; Dawson and Maloney 2017). Of note, the fur mass and fur bulk density were the same in the reds and greys; the high fibre density in the red kangaroo was balanced by layers of significantly longer outer and under fur fibres in the grey kangaroo, resulting in equivalent insulation. Together these findings highlight the significance of the crimped and upright fibres in limiting the penetration of radiation into the coat of the red kangaroo. The overall result was similar, with a relatively low heat load from incident solar radiation penetrating the backcoats in both of these arid-living kangaroos.

A curious result was the decrease in penetration that occurred with an increase in wind speed. The biophysics behind the changes in the insulation that came about because of changes in the air boundary layer, and even the coat itself, with an increase in wind speed is well understood, as outlined above. But a photon striking a coat element and being either reflected or absorbed should be independent of wind speed. A decrease in penetrance with an increase in wind speed has been reported previously, but not explained (Dawson et al. 2014). It is likely that similar mechanisms are at play to the effect of wind on the insulation of the coat. When wind speed increases, it removes the air boundary layer, and so decreases the overall insulation. Above a certain wind speed, the air within the fibres of the coat becomes disturbed, and the wind “cuts” into the coat (Tregear 1965), and also depresses the coat. A couple of things happen in the process. Firstly, the depression of the coat will effectively increase the fibre density, and also decrease the lie angle. Both of those factors, in turn, impact the penetration of radiation into the coat. A higher fibre density makes it more likely that a photon will strike a coat element closer to the coat surface, and a decrease in lie angle similarly presents more surface area of each element to incident photons. In contrast, it has been suggested that some species can physiologically adjust the coat to maximise penetrance in cold conditions. When they sun-bask in cold conditions, the Djungarian hamster and dunnart are thought to “part” the fur allowing penetration of solar radiation to the darker underfur where it is absorbed (Geiser et al. 2016; Wacker et al. 2016). Basking can significantly reduce the costs of thermoregulation and rewarming from torpor in those species.

A question arises as to whether the differences in coat structure of the two kangaroo species are part of adaptations to living in the same habitat? The answer is that they effectively do not live in the same habitat, and it is likely that coat colouring is primarily cryptic in function. Through most of their extensive ranges, these two species do not overlap. Broadly, red kangaroos inhabit the arid interior of Australia, an area that consists of sparsely vegetated desert through to arid grassland and saltbush / bluebush shrublands in more southern regions. In most of that habitat, shade is scarce with only patches of stunted trees, often acacias. The habitat preferred by western grey kangaroo is dominated by low woodland, often with an understory of shrubs. In dry landscapes where the two kangaroo species do overlap, habitat is patchy, and specialisation in habitat use is apparent (McCullough and McCullough 2000; Dawson 2012).

The summer kangaroo coats that we examined came from an area that encompasses these habitat features. This site on Fowlers Gap Arid Zone Research Station was also used by McCarron (1990) in investigations into habitat use by kangaroos. The area (18.2 km^2^) was open arid shrub/grassland on red soil plains. A large creek with dense stands of River red gums (*Eucalyptus camaldulensis*) traversed the area and patches of acacia thicket (*Acacia victoriae*) occurred along scattered drainage lines associated with a creek. During McCarron’s study, an estimated 2400 kangaroos were on the site, of which 90% were reds. The distribution of species was uneven, and in open shrub/grassland the ratio of red kangaroos to grey kangaroos was 11, whereas it declined to 4 around the acacia thickets, and to 2 along the creek line. Because the bulk of adult red kangaroos and grey kangaroos are largely sedentary (Dawson 2012), these results reflect the characteristics of the home range habitats of the species. During his study, McCarron (1990) noted that the weekly home range of both species was less than 300 ha. Broadly, kangaroos are nocturnal feeders (Dawson 2012) and so the core of the home range, around 34 ha, was indicative of their regular daytime resting sites. For the red kangaroo in hot summers those sites were associated with scattered small trees or clumps of larger bushes. Individuals moved from their resting places in the late afternoon to feed on the dry open grassland, returning to their resting sites not long after dawn. In cooler seasons, the core areas were generally more remote from vegetation cover. Behavioural and feeding patterns for the western grey were similar, but the core areas were focused on bands of acacia thicket, and that remained so in the cooler season. The greys also generally remained within 0.5–2 km of a drinking source, while the reds moved much further from sources of water. A suite of ecophysiological adaptations in the red kangaroo facilitates their lower dependence on surface water and allows them to forage over the arid shrub/grasslands (Dawson 2012) more extensively. The modification of their fur fibres that we describe here appears to be a further facet of such adaptation in that it markedly ameliorates solar heat load while providing cryptic colouration. A pertinent case in point here is that the blue morph (light grey) of the red kangaroo is restricted mainly to the smaller females in the saltbush/bluebush shrublands in semi-arid southern Australia. Though the reflectance of the fur of the blue morph is marginally lower than for the red morph, heat flow patterns do not differ significantly (Dawson and Maloney 2004; Dawson and Maloney 2017).

The significance of cryptic colouration is highlighted by flight responses, presumably developed as an adaptation to predation (Dawson 2012). Feeding aggregations of mixed species of kangaroos occurred in the vicinity of drainage lines. When these groups were startled, the greys quickly made for the cover provided by the acacia thicket and the scrub undergrowth. If pressed, they used their mobility through cover and over broken ground to be soon out of harm's way. Red kangaroos, on the other hand, took off away from treed areas and quickly entered the open shrub/grasslands. If they were forced toward the creek line, they generally went straight through and out the other side. It seems that the red kangaroo deals with predation by getting into the open where they can rely on their superior speed and crypsis with their environment.

The principal insight emerging from our study of the summer furs of differing coloured species of kangaroos from a hot arid environment is the complexity involved in achieving competing aims. It has been considered that the lighter coloured, more reflective, coat of the red kangaroo is more of a thermoregulatory benefit in confronting solar heat loads than is the darker coat of the grey kangaroo. But the lighter colour should result in deeper penetration of solar radiation into the lighter fur. Functional parity in solar heat loads, however, was achieved between red and grey kangaroos through mitigating modifications in fibre density and fibre structural features that are not present in the furs of other kangaroos. It seems that crypsis has been a major participant in coat colour evolution in kangaroos.

## Figures and Tables

**Fig. 9:**
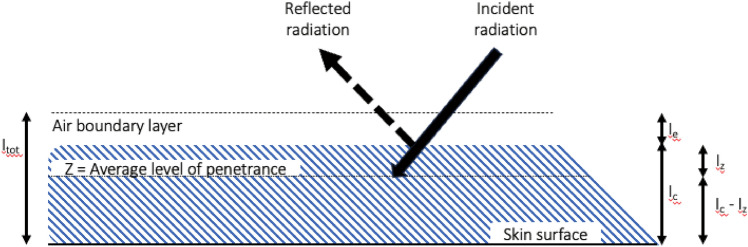
Simplified model of the fate of solar radiation that is incident on mammalian fur. Incident radiation passes through the air boundary layer and can be either reflected back to the environment (dashed arrow) or absorbed at various levels within the coat. The average penetrance level is denoted by *Z*, and the insulation between there and the coat surface is *I*_*z*_. The total insulation of the pelt (*I*_tot_) is given by the insulation provided by the coat (*I*_*c*_) plus the air boundary layer (*I*_*e*_); the latter is influenced by wind speed

## Data Availability

The raw data are available from the authors on request.

## Appendix

See Fig. 9.
